# Acknowledgement of manuscript reviewers 2014

**DOI:** 10.1186/s12939-015-0141-7

**Published:** 2015-02-21

**Authors:** Ana Lorena Ruano, Efrat Shadmi, Leiyu Shi

**Affiliations:** International Journal for Equity in Health, ᅟ, ᅟ

## Abstract

The editors of *International Journal for Equity in Health* would like to thank all our reviewers who have contributed to the journal in Volume 13 (2014).

**Jens Aagaard-Hansen**

Denmark

**Muhammad Ahmed Abdullah**

Pakistan

**Ibraheem Abdulraheem**

Nigeria

**Gilbert Abiiro**

Ghana

**Ron Acierno**

United States of America

**Suneth Agampodi**

Sri Lanka

**Jun Aida**

United Kingdom

**Shamima Akhter**

Bangladesh

**Vassilis Aletras**

Greece

**Sara Allin**

Canada

**Djesika Amendah**

Kenya

**Rachel M Amiya**

Japan

**Sohail Amjad**

Pakistan

**Mary Amoakoh-Coleman**

Ghana

**Corinne Armstrong**

United Kingdom

**Genevieve Aryeetey**

Ghana

**Farah Asna Ashari**

Germany

**Gizachew Assefa Tessema**

Ethiopia

**Stephen Ayisi Addo**

Ghana

**Mona Backhans**

Sweden

**Aristide Bado**

Burkina Faso

**Juan Baeza**

United Kingdom

**Teresa Bago D'Uva**

Netherlands

**Ross Bailie**

Australia

**Petra Baji**

Hungary

**Mate Aadam Balazs**

Hungary

**Karen Bandeen-Roche**

United States of America

**Rita Barata**

Brazil

**Orna Baron-Epel**

Israel

**Robert Basaza**

Uganda

**Irmgard Bauer**

Australia

**Charles Begley**

United States of America

**Morton Beiser**

Canada

**Ana Paula Belon**

Canada

**Anita Bercovitz**

United States of America

**Lennart Bogg**

Sweden

**Guglielmo Bonaccorsi**

Italy

**Billie Bonevski**

Australia

**Igna Bonfrer**

Netherlands

**Carme Borrell**

Spain

**Paula Braveman**

United States of America

**Manuel Bravo**

Spain

**Mayer Brezis**

Israel

**Mark Brough**

Australia

**Barbara Bucki**

France

**Sarah Burgard**

United States of America

**Gerard Bury**

Ireland

**Peter Byass**

Sweden

**Lourdes Cantarero Arévalo**

Denmark

**Elaine Carnegie**

United Kingdom

**Miriam Carter**

Switzerland

**Anna Cristina Carvalho**

Italy

**Monica Castro**

Brazil

**Francesca Cavallaro**

United Kingdom

**Shivani Chandra**

India

**Hsien-Yen Chang**

United States of America

**Shu-Sen Chang**

Hong Kong

**Shiau-Fang Chao**

Taiwan

**Katrina Charles**

United Kingdom

**Thomas Charters**

Canada

**Nearkasen Chau**

France

**Nita Chaudhuri**

France

**Brian Chen**

United States of America

**Wen Chen**

China

**Yingyao Chen**

China

**Lingguo Cheng**

United States of America

**Cecile Cherrier**

France

**Eun-Kyong Choi**

United States of America

**Ekta Choudhary**

United States of America

**Terkel Christiansen**

Denmark

**Roger Chung**

Hong Kong

**James Ciera**

Kenya

**Ron Cisler**

United States of America

**Anna-Britt Coe**

Sweden

**Donald Cole**

Canada

**Christopher Colvin**

South Africa

**Isabel Correia**

Portugal

**Neil Coulson**

United Kingdom

**Jonathan Cylus**

United Kingdom

**Phyllis Dako-Gyeke**

Ghana

**Gianfranco Damiani**

Italy

**Irene Dankwa-Mullan**

United States of America

**Deborah De Moortel**

Belgium

**Treena Delormier**

United States of America

**Anahit Demirchyan**

Armenia

**Kebede Deribe**

Ethiopia

**Michele Devlin**

United States of America

**Shafik Dharamsi**

Canada

**Michelle Digiacomo**

Australia

**Mamuka Djibuti**

Georgia

**Omara Dogar**

United Kingdom

**Antonia Domingo-Salvany**

Spain

**Christopher Dowrick**

United Kingdom

**Lesley Doyal**

United Kingdom

**Esther Dungumaro**

Tanzania

**Mark Dusheiko**

Switzerland

**Daniel Dutton**

Canada

**Marina Economou**

Greece

**Andrew Edmonds**

United States of America

**Frida Eek**

Sweden

**Mary Egan**

Canada

**Bolaji Egbewale**

Nigeria

**Heather Eicher-Miller**

United States of America

**Björn Ekman**

Sweden

**Eric Emerson**

United Kingdom

**Maria Emmelin**

Sweden

**Jorge Escobedo**

Mexico

**Marie-Louise Essink-Bot**

Netherlands

**Rebecca Evans**

Australia

**Stephanie Farquhar**

United States of America

**Stefano Federici**

Italy

**Andrea Feigl**

United States of America

**Limin Feng**

China

**Shingairai Feresu**

United States of America

**Astrid Fink**

Germany

**Kevin Fiscella**

United States of America

**Steffen Flessa**

Germany

**Walter Flores**

Guatemala

**Ameneh Setareh Forouzan**

Iran

**Sari Fröjd**

Finland

**Chuangluan Fu**

China

**John Furler**

Australia

**Charles Gallia**

United States of America

**Ana Gama**

Portugal

**Hongxia Gao**

China

**Deirdre Gartland**

Australia

**Tiffany Gary-Webb**

United States of America

**Tony Gatrell**

United Kingdom

**Usha George**

Canada

**Claudia Geue**

United Kingdom

**Katrien Ghoos**

Thailand

**John Gilroy**

Australia

**Beatriz Gl Valcarcel**

Spain

**Lieve Goemn**

Belgium

**Fidel Gonzalez**

United States of America

**George Gotsadze**

Georgia

**Lorraine Greaves**

Canada

**Emily Gustafsson-Wright**

United States of America

**Clement Gwede**

United States of America

**Michael Hall**

New Zealand

**Tomoya Hanibuchi**

Japan

**Lisa Harryson**

Sweden

**Nancy Haselow**

United States of America

**Seyed Saeed Hashemi Nazari**

Iran

**Michael Hawkes**

Canada

**Jean-Luc Heeb**

Switzerland

**Gerardo Heiss**

United States of America

**Marion Hitzeroth**

Germany

**Ayako Hiyoshi**

Sweden

**Anders Hjern**

Sweden

**Paula Holland**

United Kingdom

**Rolf Holle**

Germany

**Monjurul Hoque**

South Africa

**Mahabub Hossain**

Bangladesh

**Mary Howard**

United States of America

**Patricia Hudelson**

Switzerland

**Hans Husum**

Norway

**Lawrence Ikamari**

Kenya

**Victor Inem**

Nigeria

**Dena Jaffe**

Israel

**Kari Jarvinen**

Australia

**Saroj Jayasinghe**

Sri Lanka

**Weiyan Jian**

China

**Lei Jin**

Hong Kong

**Thomas Johnson**

Hong Kong

**Julia Jordan-Zacgert**

United States of America

**Hyun-Woo Joung**

United States of America

**Sol Juárez**

Sweden

**Cigdem Kagitcibasi**

Turkey

**Dimitra Kamboukos**

United States of America

**Aaron Katz**

United States of America

**Anne Kavanagh**

Australia

**Margaret Kelaher**

Australia

**Elissa Kennedy**

Australia

**Ernest Kenu**

Ghana

**Janko Kersnik**

Slovenia

**Zeinab Khadr**

Egypt

**Attia Khan**

Pakistan

**Ali S Khashan**

Ireland

**Hussein Lesio Kidanto**

Tanzania

**Holly Kiger**

United States of America

**Hongsoo Kim**

Korea, South

**Nicholas King**

Canada

**Sylvia Kirchengast**

Austria

**France Kittel**

Belgium

**Jenny Knight**

Australia

**Naoru Koizumi**

United States of America

**Peter Kolarcik**

Slovakia

**Tulio Konstantyner**

Brazil

**Rosemary Korda**

Australia

**Alfgeir Logi Kristjansson**

United States of America

**Joan Kub**

United States of America

**Yadlapalli Kusuma**

India

**Dejian Lai**

United States of America

**Mitja Lainscak**

Slovenia

**Chris Lalonde**

Canada

**Evelina Landstedt**

Sweden

**Andrew Larner**

United Kingdom

**Jorgen Lauridsen**

Denmark

**Barry Lavallee**

Canada

**Julian Le Grand**

United Kingdom

**Lydie Lebrun-Harris**

United States of America

**Albert Lee**

Hong Kong

**Jee Hye Lee**

Korea, South

**Helena Legido-Quigley**

United Kingdom

**Carolyn Lester**

United Kingdom

**Lawrence Leung**

Canada

**Hagai Levine**

Israel

**Hailun Liang**

United States of America

**Julie Lifshay**

United States of America

**Laurie Lindamer**

United States of America

**Jutta Lindert**

Germany

**Martin Lindstrom**

Sweden

**Hong Liu**

China

**Lisa Lix**

Canada

**Rickard Ljung**

Sweden

**Bebe Loff**

Australia

**Ana Lorena Ruano**

Norway

**Marta Losa Iglesias**

Spain

**Siaka Lougue**

South Africa

**David Lounsbury**

United States of America

**Peiyi Lu**

China

**Jiehua Lu**

China

**Wei Luo**

United States of America

**Kristine Lykens**

United States of America

**Jane Macha**

Tanzania

**Andrea Madarasova Geckova**

Slovakia

**Ferdinand Maduka-Okafor**

Nigeria

**Netta Mäki**

Finland

**Vilija Malinauskiene**

Lithuania

**Davide Malmusi**

Spain

**Fred Martineau**

United Kingdom

**Saidur Mashreky**

Bangladesh

**Mitsuaki Matsui**

Japan

**Pallab Maulik**

India

**Annette Maxwell**

United States of America

**Maureen Mayhew**

Canada

**Maurice Mbago**

Tanzania

**Tim Mccreanor**

New Zealand

**Joan Mcdowell**

United Kingdom

**John Mckie**

Australia

**Rachael Mclean**

New Zealand

**Graham Meadows**

Australia

**Maria Gabriella Melchiorre**

Italy

**Devidas Menon**

Canada

**Christopher Millett**

United Kingdom

**Helen Miltiades**

United States of America

**Kaelan Moat**

Canada

**Tirelo Modie-Moroka**

Botswana

**Susanne Moebus**

Germany

**Victoria Monchuk**

United States of America

**Paola Andrea Mosquera**

Colombia

**Jin Mou**

Canada

**Cheryl Moyer**

United States of America

**Fatima Mukhtar**

Pakistan

**Moses Kinyanjui Muriithi**

Kenya

**Namuunda Mutombo**

Kenya

**Adamson Muula**

Malawi

**Sumal Nandasena**

Sri Lanka

**Solange Nappo**

Brazil

**Benjamin Nganda**

Kenya

**Ellen Nolte**

United Kingdom

**Justice Nonvignon**

Ghana

**Jacob Novignon**

Ghana

**Thomas Novotny**

United States of America

**Behdin Nowrouzi**

Canada

**Solomon Nyame**

Ghana

**Walter Obiero**

United States of America

**Fidelia Ohemeng**

Ghana

**Theodora Okeke**

Nigeria

**Oystein Evjen Olsen**

Norway

**Obinna Onwujekwe**

Nigeria

**Arash Ordookhani**

United States of America

**Gaby Ortiz**

Norway

**Lars Osberg**

Canada

**Stanislava Otasevic**

Serbia

**Maharouf Oyolola**

Kenya

**Mojgan Padyab**

Sweden

**Donatella Panatto**

Italy

**Supasit Pannarunothai**

Thailand

**Guillermo Paraje**

Chile

**Alison Parken**

United Kingdom

**Shilpa Patel**

United Kingdom

**Will Perez**

Nicaragua

**Amaya Perez-Brumer**

United States of America

**Karen Pesse**

Ecuador

**David Peters**

United States of America

**Tarryn Phillips**

Australia

**Jane Phiri**

South Africa

**Fernando Poblete**

Chile

**Tonia Poteat**

United States of America

**Gawaine Powell Davies**

Australia

**Naomi Priest**

Australia

**Ivan Pristas**

Croatia

**Ritu Priya**

India

**Sarah Prout**

Australia

**Dongfu Qian**

China

**Yulin Qiu**

China

**Rosanna Quattrin**

Italy

**Amelie Quesnel-Vallee**

Canada

**Kumanan Rasanathan**

United States of America

**J.J. Rasker**

Netherlands

**Mihi Ratima**

New Zealand

**Maija Reblin**

United States of America

**Jessica Reid**

Canada

**Sijmen A. Reijneveld**

Netherlands

**Gemma Renart-Vicens**

Spain

**Mohsen Rezaeian**

Iran

**Joerg Richter**

Norway

**Jan Rigby**

Ireland

**Marisol Rodriguez**

Spain

**Maica Rodriguez**

Spain

**Mikael Rostila**

Sweden

**Kevin Rowley**

Australia

**Mariano Salazar**

Sweden

**Nouha Saleh Stattin**

Sweden

**Miguel San Sebastian**

Sweden

**María Sánchez-Domínguez**

Sweden

**Franziska Satzinger**

Germany

**Ola Didrik Saugstad**

Norway

**Erik Schokkaert**

Belgium

**Lisa Schölin**

United Kingdom

**Vera Schölmerich**

Netherlands

**Sidney Schuler**

United States of America

**Efrat Shadmi**

Israel

**Anthony Shakeshaft**

Australia

**Guoliang Shi**

China

**Lizheng Shi**

United States of America

**Olga Siskou**

Greece

**Catherine Smith**

Australia

**Patricia M Smith**

Canada

**Mariana Socal**

United States of America

**Johannes Sommerfeld**

Switzerland

**Michela Sonego**

Spain

**Rene Soria Saucedo**

Bolivia

**Kyriakos Souliotis**

Greece

**Ireneous N. Soyiri**

Malaysia

**Stuart Speedie**

United States of America

**Joshua Ssebunnya**

Uganda

**Maisha Standifer**

United States of America

**Stephen Stansfeld**

United Kingdom

**Jennifer Stewart Williams**

Australia

**Alexandrina Stoyanova**

Spain

**Jackie Street**

Australia

**Canlan Sun**

United States of America

**Qiang Sun**

China

**Wannapa Suwonkerd**

Thailand

**S.Jamaledin Tabibi**

Iran

**Simbarashe Takuva**

South Africa

**Makram Talih**

United States of America

**Wassim Tarraf**

United States of America

**Francis Tayie**

United States of America

**Angela Testi**

Italy

**Rachelle Theise**

United States of America

**Heike Thiel De Bocanegra**

United States of America

**Margaret Thorogood**

United Kingdom

**Christiana Titaley**

Indonesia

**Jenny Torssander**

Sweden

**Marie Tournier**

France

**Shin-Ichi Toyabe**

Japan

**Reshma Trasi**

India

**Claudia Travassos**

Brazil

**Henrie Treadwell**

United States of America

**Vladimir Trkulja**

Croatia

**Cleon Tsimbos**

Greece

**Junya Tsutsui**

Japan

**Reginald Tucker-Seeley**

United States of America

**Peter Tugwell**

Canada

**Ho-Jui Tung**

Taiwan

**Yu-Chi Tung**

Taiwan

**Nkolika Uguru**

Nigeria

**Mirjana Ule**

Slovenia

**Rosa M. Urbanos-Garrido**

Spain

**Olalekan Uthman**

Niger

**Masoud Vaezghasemi**

Sweden

**Sjaak Van Der Geest**

Netherlands

**Philip Van Der Wees**

Netherlands

**Kjetil A. Van Der Wel**

Norway

**Hans Van Kippersluis**

Netherlands

**Ronan Van Rossem**

Belgium

**Christophe Vanroelen**

Belgium

**Felipe Vasquez Lavin**

Chile

**Michel Vernay**

France

**Georgia Verropoulou**

Greece

**Joan R Villalbí**

Spain

**Carmen Vives-Cases**

Spain

**Olaf Von Dem Knesebeck**

Germany

**Athanassios Vozikis**

Greece

**David Walsh**

United Kingdom

**You Wang**

China

**Yumin Wang**

China

**Jian Wang**

China

**Li Wang**

Canada

**Jonas Wastesson**

Sweden

**Jacqui Webster**

Australia

**Andrea Whittaker**

Australia

**Warren Wilson**

Canada

**William Chi Wai Wong**

Hong Kong

**Jianjun Wu**

China

**Zhuochun Wu**

China

**Hongwei Xu**

United States of America

**Zhijun Yang**

China

**Elias Yesuf**

Ethiopia

**Denise Zabkiewicz**

Canada

**Jennifer Zeitlin**

France

**Kangzhi Zhang**

China

**Yuejen Zhao**

Australia

**Chengchao Zhou**

China

**Jane Zhu**

United States of America

**Junya Zhu**

United States of America

**Oscar Zurriaga**

Spain

